# The relationship between psoriasis and vitiligo: From a comprehensive study

**DOI:** 10.1111/srt.13868

**Published:** 2024-07-19

**Authors:** Junchen Li, Qian Shen, Yingdong Wang, Chenqi Guo, Yuxiao Ma, Yu Zhang

**Affiliations:** ^1^ Graduate School Tianjin University of Traditional Chinese Medicine Tianjin China; ^2^ Guang'anmen Hospital China Academy of Chinese Medical Sciences Beijing China; ^3^ Dermatology Department Tianjin Academy of Traditional Chinese Medicine Affiliated Hospital Tianjin China

**Keywords:** causation, correlation, Mendelian randomization, psoriasis, vitiligo

## Abstract

**Background:**

Both psoriasis and vitiligo are autoimmune skin diseases. Previous observational studies have indicated a relationship between the two conditions, and simultaneous onset of both diseases poses increased health risks to patients. However, limited research has explored the causal relationship between psoriasis and vitiligo.

**Objectives:**

To investigate whether a causal association exists between psoriasis and vitiligo.

**Methods:**

A case of Chinese patients diagnosed with psoriasis and vitiligo has been reported. Transcriptome sequencing was performed on normal, psoriasis, vitiligo, and co‐morbid skin tissues of the patients, and single‐cell transcriptome sequencing was conducted on the co‐morbid skin tissues. A comprehensive Mendelian randomization analysis of Genome‐wide association studies (GWAS) was performed on a cohort of 261 018 European individuals with psoriasis from the IEU Open GWAS Project and vitiligo from the National Institutes of Health (NIH) Database of Genotypes and Phenotypes.

**Results:**

Case report and transcriptome results showed that skin tissue with vitiligo combined with psoriasis exhibited both vitiligo and psoriasis. Single‐cell transcriptome sequencing results showed that in comparison to normal skin and psoriatic skin, the proportions of CD8+ T cells, natural killer cells, naive T cells, T helper cells 17, regulatory T cells, conventional type 1 dendritic cells, Conventional type 2 dendritic cells, and plasmacytoid dendritic cells were all increased in skin tissue with vitiligo combined with psoriasis. Mendelian randomization analysis included 4510 patients with psoriasis and 4680 patients with vitiligo. The results showed no causal relationship between vitiligo and psoriasis in the forward direction (*p* = 0.192; odds ratio [OR], 1.059; 95% confidence interval [CI], 0.971–1.155) or in the reverse direction (*p* = 0.459; OR, 0.927; 95% CI, 0.757–1.134).

**Conclusions:**

This study suggests that the association between psoriasis and vitiligo may be closely related to immunity, however, Mendelian randomization studies do not support a causal relationship. These findings hold significant implications for clinicians aiming to enhance their understanding and treatment approaches for psoriasis and vitiligo.

## 1 INTRODUCTION

Psoriasis and vitiligo, both autoimmune skin diseases, have been recognized to share a relationship since 1890. Early observations suggested that some patients exhibited psoriasis and vitiligo lesions in the same areas. The coexistence of these conditions, known as comorbidity, is a significant phenomenon. However, some scholars argue that comorbidity may simply be a coincidental overlap without a substantial underlying connection between the two diseases.^1^ In recent years, several clinical review studies have indicated an association between psoriasis and vitiligo.^2^, ^3^, ^4^ There is an increasing consensus suggesting that patients with both conditions are at a higher risk of having a family history of cardiovascular disease, experiencing inflammatory responses, and developing other autoimmune diseases.^5^, ^6^, ^7^ Observational studies have been conducted to explore the relationship between psoriasis and vitiligo, but these studies face challenges such as confounding factors and reverse causality, which can introduce bias. Consequently, the underlying mechanism and causal relationship between psoriasis and vitiligo remain unclear. Therefore, this study focuses on an adult male patient who solely has a comorbidity of psoriasis and vitiligo without any other relevant medical history. A transcriptome study was conducted to investigate the association between the two diseases, and Mendelian randomization analysis was employed to provide more reliable results compared to conventional randomized controlled trials.^8^ This study aims to examine the bidirectional causal relationship between psoriasis and vitiligo, shedding light on the connection between these conditions.

## 2 METHODS

This study was approved by the Ethics Committee of the Affiliated Hospital of Tianjin Academy of Traditional Chinese Medicine (approval number: LLYK2020‐21). Patients participating in this study voluntarily signed the informed consent. The original research conducted for this study has obtained ethical approval and ensured that participants provided informed consent. All experiments in this study complied with the Declaration of Helsinki and were conducted in accordance with relevant guidelines and regulations.

### 2.1 Case report

The study included a 32‐year‐old male patient who had comorbid psoriasis and vitiligo. The patient's inclusion was based on the presence of both psoriasis and vitiligo manifestations in the histopathological sections of the skin. The patient had a history of psoriasis for 5 years and vitiligo for 30 years, with no other relevant medical history. Prior to receiving treatment, the patient underwent various tests including blood routine, urine routine, biochemical, and immunological tests. Dermoscopy, Wood lamp examination, and skin CT scans were performed on normal skin (C), psoriasis skin (P), vitiligo skin (V), and skin areas with both vitiligo and psoriasis (VP). In the outpatient operating room, superficial skin tissue biopsies were taken from patients C, P, V, and VP. The biopsied tissue was rapidly rinsed in pre‐cooled RNase free water, frozen in liquid nitrogen, and transported to the laboratory under low‐temperature conditions for transcriptome sequencing. Additionally, single‐cell transcriptome sequencing was performed on the skin tissue with both vitiligo and psoriasis.

### 2.2 Mendelian randomization study

The psoriasis data used in this study was obtained from the dataset ID finn‐b‐L12_PSORIASIS in the IEU Open GWAS Project. This dataset represents a European population and includes 4510 psoriasis cases and 212 242 controls. For vitiligo data, the datasets with IDs phs000224.v1.p1, phs000224.v2.p1, and phs000224.v3.p1 from the NIH Database of Genotypes and Phenotypes were utilized. These datasets also consist of a European population and contain 4680 vitiligo cases and 39 586 controls. The details of this vitiligo dataset have been published previously.^9^ Mendelian randomization studies were conducted using single‐nucleotide polymorphisms (SNPs) as instrumental variables (IVs). SNPs with a significance threshold of *p* ≤ 5 × 10^−8^ and a minor allele frequency ≥3% were included, and SNPs with reported loci overlap or linkage disequilibrium R2 < 0.001 were excluded during screening. In our clumping process, we used a linkage disequilibrium window size of 10 000 kb. To minimize bias resulting from weak IVs, further screening was performed with a criterion of *F* >  10, and palindromic SNPs were also excluded to obtain the final set of IVs. The canonical Mendelian randomization analysis was conducted following the Strengthening the Reporting of Observational Studies in Epidemiology using Mendelian Randomization (STROBE‐MR) guidelines.^10^ The analysis process adhered to three assumptions of Mendelian randomization studies: (1) the correlation assumption, where the SNP is strongly associated with the exposure; (2) the exclusion assumption, where the SNP is unrelated to the outcome; and (3) the independence assumption, where the SNP is unrelated to confounding factors. Various methods, including inverse variance weighting (IVW), weighted median (WM), MR‐Egger, weighted model, and simple model, were used to estimate the causal relationship between the exposure (Vitiligo/Psoriasis) and outcome (Psoriasis/Vitiligo). Heterogeneity was tested using Cochran's Q, and pleiotropy was examined through MR‐Egger regression of intercept values. Additionally, PhenoScanner (http://www.phenoscanner.medschl.cam.ac.uk/) was utilized to detect links between genes and other diseases, aiding in the identification and exclusion of gene pleiotropy. All analyses were performed using R 4.1.2 software, incorporating packages such as “MR‐PRESSO,” “Two‐Sample MR,” and “MR.RAPS.”

## 3 RESULTS

### 3.1 Case report

**FIGURE 1:**
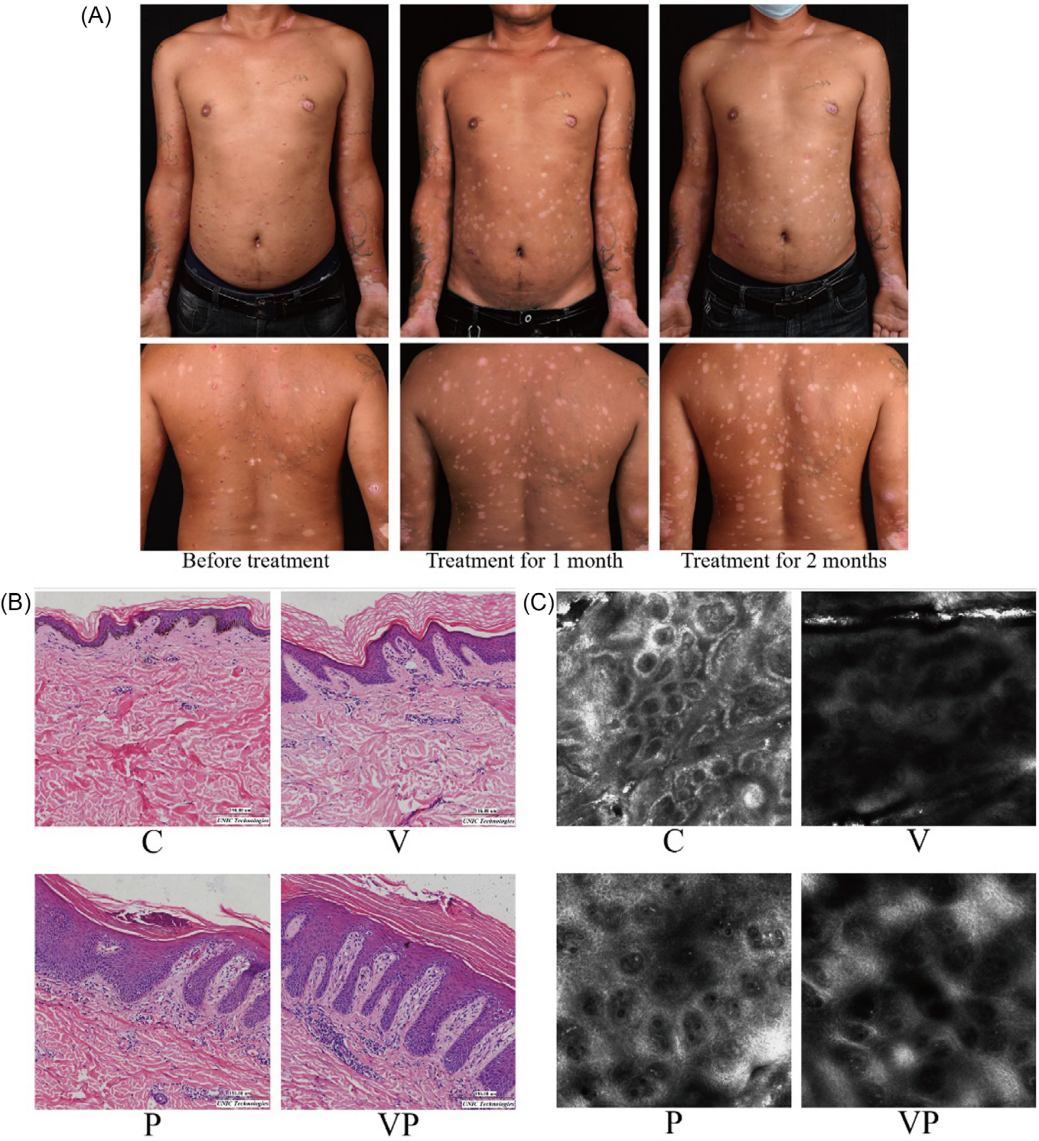
Skin conditions in comorbid patients. (A) The patient's clinical photograph; (B) Results of the skin CT examination; (C) Histopathological results.

The physical examination of the patient revealed the presence of multiple erythema, scales, and bright red depigmented spots with blurred edges prior to treatment. After 1 month of conventional therapy, the erythema on the trunk became less intense, and the scales disappeared. Some psoriasis lesions subsided, leaving behind bright red depigmented spots with fuzzy edges. The original bright red depigmented spots became less vibrant, and the edges became clearer. After 2 months of treatment, the erythema and scales disappeared from the entire body except for the extremities. The remaining depigmented spots and the original depigmented spots turned white with clear edges, and pigment islands became visible inside. Figure 1A illustrates the patient's clinical photograph. Before treatment, there were no notable abnormalities found in the complete blood count, routine urinalysis, biochemical studies, and immunological examinations. After treatment, these examinations were rechecked, and no significant abnormalities were observed. Dermoscopy revealed the following characteristics: in psoriasis lesions, uniformly distributed punctate blood vessels on a red background, covered with scales; in vitiligo lesions, residual pigment around the hair follicles and reticular hypopigmentation; in comorbid psoriasis and vitiligo lesions, a combination of the above‐mentioned characteristics of both V and P diseases. Wood lamp examination showed that in psoriasis lesions, there were no specific manifestations; in vitiligo lesions, the skin lesion appeared off‐white with unclear boundaries, and the area of skin lesions under the Wood lamp was larger than the visible area; in comorbid psoriasis and vitiligo lesions, it exhibited characteristics of both V and P diseases simultaneously. Skin CT examination revealed the following findings: in P lesions, hyperkeratosis, parakeratosis, upper edge of dermal papilla, capillary dilation, and congestion; in V lesions, a significant reduction in basal layer pigment and absence of basal cell ring; in VP lesions, it demonstrated characteristics of both V and P diseases. Figure 1B presents the results of the skin CT examination. Histopathological examination showed that in P lesions, there was hyperkeratosis with insufficient keratosis, Munro microabscess composed of neutrophils in the epidermis, thickening of the spinous layer, thinning of the granular layer, elongation of the epidermal process with wider ends, and neutrophil infiltration in the superficial dermis. In V lesions, there was a decrease in basal layer melanocytes and a significant reduction in melanin granules. In VP lesions, the pathological changes observed were a combination of the aforementioned characteristics of both V and P diseases. The histopathological results are illustrated in Figure 1C.

**FIGURE 2:**
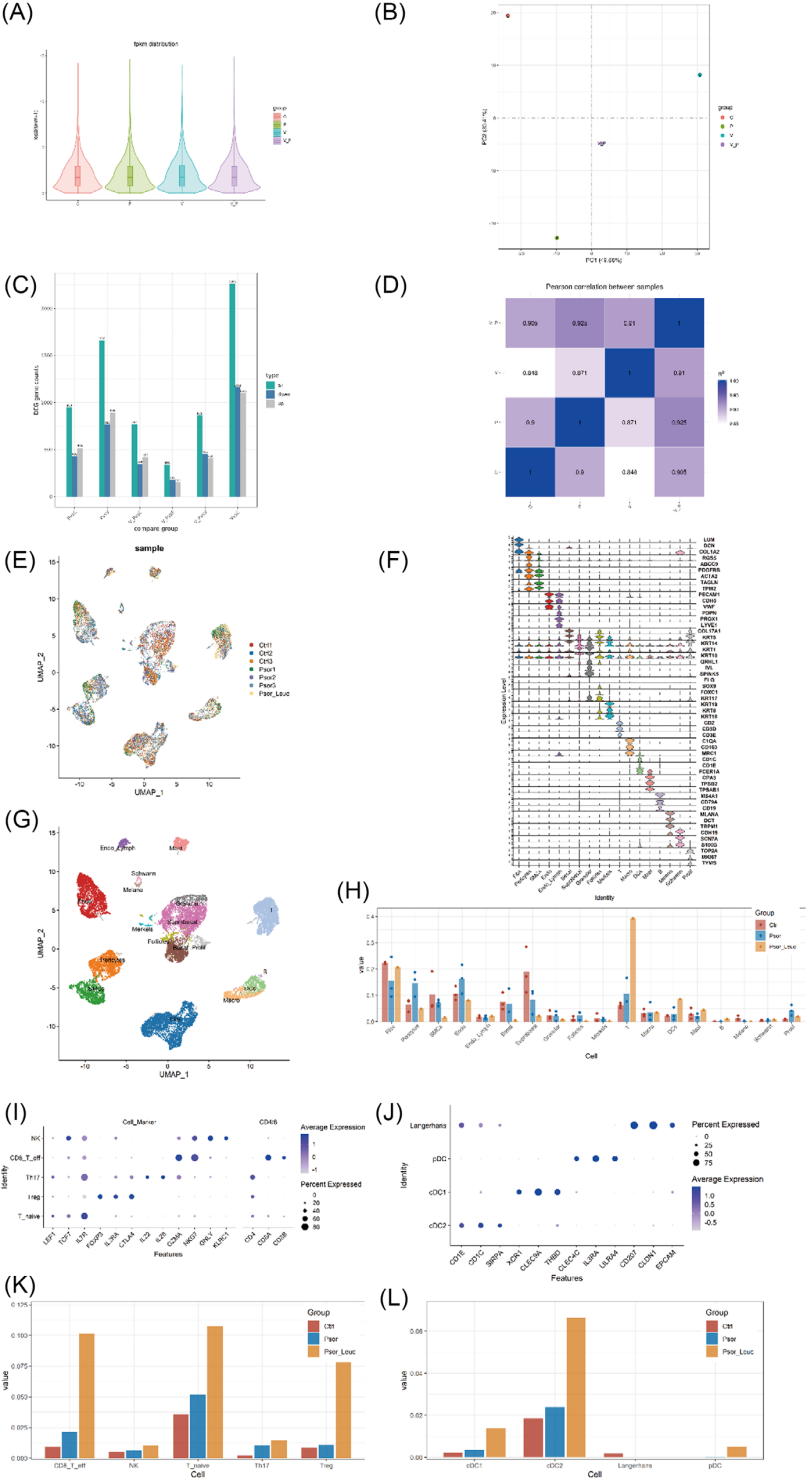
Transcriptome results. (A) Gene expression levels of each sample; (B) resulting in the PCA diagram; (C) number of differential genes for each comparison combination; (D) correlation coefficient diagram of each sample; (E) clustering results of cell subsets in each sample; (F) expression of marker genes in different cell subsets; (G) annotated cell subsets; (H) statistical results of the annotated cell subpopulations; (I) expression of marker genes in different cell subsets of T cells; (J) expression of marker genes in different cell subsets of DC cells; (K) statistical results regarding the further annotated numbers of each cell subpopulation of T cells; (L) statistical results regarding the further annotated numbers of each cell subpopulation of DC cells.

Transcriptome sequencing was conducted on the skin tissues of C, P, V, and VP, and the gene expression levels of each sample are depicted in Figure 2A. The overall gene expression levels in the four tissues appeared similar and comparable. Principal component analysis was employed based on the gene expression levels of each sample, resulting in the PCA diagram shown in Figure 2B. The distance between C and V, P, and VP was significantly distant, indicating substantial dissimilarity between normal skin tissue and diseased skin tissue. VP was positioned between V and P on the PCA plot, suggesting that at the transcriptome level, VP exhibits gene expression characteristics of both V and P. The number of differential genes for each comparison combination is presented in Figure 2C. The number of differential genes observed between VP and P, as well as between VP and V, was higher compared to the number of differential genes between P and V. This finding indicates that the gene expression profile of VP is similar to both P and V. The correlation coefficient diagram of each sample is displayed in Figure 2D, revealing a close relationship between VP, P, and V.

Single‐cell transcriptome sequencing was carried out on skin tissues that exhibited comorbidity of vitiligo and psoriasis. In addition, single‐cell transcriptome sequencing data from three cases of normal skin tissues and three cases of psoriasis skin tissues from the GSE162183 dataset were included for analysis. The clustering results of cell subsets in each sample are presented in Figure 2E. Cell markers from literature and databases were used for annotation, and the expression of marker genes in different cell subsets is illustrated in Figure 2F. The annotated cell subsets are depicted in Figure 2G. Statistical results of the annotated cell subpopulations are shown in Figure 2H, categorized by sample types. Comparatively, skin with vitiligo and psoriasis exhibited increased proportions of T cells, DC cells, Mast cells, and B cells when compared to normal skin and psoriatic skin. Further annotation was performed for T cells and DC cells using cell markers from literature and databases. The expression of marker genes in different cell subsets of T cells and DC cells is shown in Figure 2I,J, respectively. Statistical results regarding the further annotated numbers of each cell subpopulation of T cells and DC cells, categorized by sample types, are displayed in Figure 2K,L, respectively. It can be observed that, in comparison to normal skin and psoriatic skin, the proportions of CD8+ T cells, natural killer cells, naive T cells, T helper cells 17, regulatory T cells, conventional type 1 dendritic cells, Conventional type 2 dendritic cells, and plasmacytoid dendritic cells were all increased in VP skin tissues.

### 3.2 Mendelian randomization study

**FIGURE 3:**
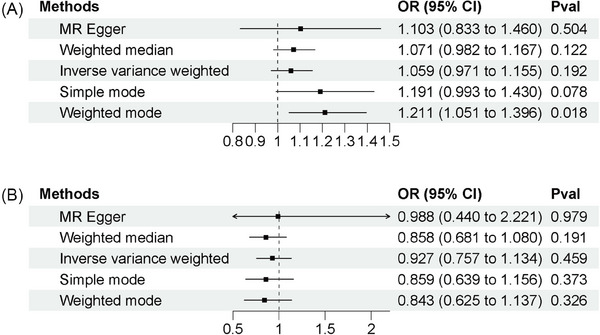
Mendelian randomization study results. (A) MR results of vitiligo and psoriasis; (B) MR results of psoriasis and vitiligo.

After a thorough screening process, we selected 17 single‐nucleotide polymorphisms as instrumental variables for the Mendelian randomization analysis of vitiligo versus psoriasis. Detailed information about these SNPs can be found in Table S1. Similarly, 5 SNPs were chosen as IVs for the Mendelian randomization analysis of psoriasis versus vitiligo, and their details are provided in Table S2. The IVW method was employed, and the results indicated that there was no evidence of a causal relationship between vitiligo and psoriasis (*p* = 0.192; odds ratio [OR], 1.059; 95% confidence interval [CI], 0.971–1.155). This finding was consistent across MR‐Egger, weighted median, simple mode, and weighted mode analyses, all of which indicated the absence of a causal relationship between vitiligo and psoriasis. The corresponding results can be observed in Figure 3A and Table 1. Similarly, the IVW results demonstrated no causal relationship between psoriasis and vitiligo (*p* = 0.459; OR, 0.927; 95% CI, 0.757–1.134). This finding was further supported by MR‐Egger, weighted median, simple mode, and weighted mode analyses, all of which provided consistent results indicating the absence of a causal relationship between psoriasis and vitiligo. The results can be visualized in Figure 3B and Table 2. Additional analyses, such as scatterplots, leave‐one‐out analysis, heterogeneity analysis, and pleiotropic analysis, were conducted to ensure the reliability of the results. Figures [Link], [Link], and Table S3 provide further details on these analyses.

**TABLE 1:** MR results of vitiligo and psoriasis.

Method	No. SNP	Beta	SE	*p‐*value	OR	OR 95% CI
MR Egger	17	0.098	0.143	0.504	1.103	0.833–1.460
Weighted median	17	0.068	0.044	0.122	1.071	0.982–1.167
Inverse variance weighted	17	0.058	0.044	0.192	1.059	0.971–1.155
Simple mode	17	0.175	0.093	0.078	1.191	0.993–1.430
Weighted mode	17	0.192	0.072	0.018	1.211	1.051–1.396

**TABLE 2:** MR results of psoriasis and vitiligo (reverse).

Method	No. SNP	Beta	SE	*p‐*value	OR	OR 95% CI
MR Egger	5	−0.012	0.413	0.979	0.988	0.440–2.221
Weighted median	5	−0.154	0.117	0.191	0.858	0.681–1.080
Inverse variance weighted	5	−0.076	0.103	0.459	0.927	0.757–1.134
Simple mode	5	−0.152	0.151	0.373	0.859	0.639–1.156
Weighted mode	5	−0.171	0.153	0.326	0.843	0.625–1.137

## 4 DISCUSSION

The clinical data and examinations conducted in this case report provide strong support for the diagnosis of psoriasis and vitiligo comorbidity. This study represents the first self‐control transcriptome sequencing investigation involving different skin tissues from patients with comorbid psoriasis and vitiligo. Additionally, it is the first single‐cell transcriptome sequencing study focusing on skin affected by both psoriasis and vitiligo. In China, there have been 45 case reports documenting psoriasis and vitiligo comorbidity (a list of pathological reports can be found in Supplementary material S1), encompassing a total of 76 patients. After excluding four patients who did not report the onset times of the two diseases, it was found that 69.4% experienced the onset of vitiligo before psoriasis, while 30.6% experienced psoriasis prior to vitiligo. The patients observed in this study exhibited vitiligo onset at the age of 2 and psoriasis onset at the age of 27. Similar clinical observations in Korea and Israel indicate that psoriasis is more likely to develop following the onset of vitiligo.^11^, ^12^ These findings imply that the presence of vitiligo creates a favorable environment for the subsequent development of psoriasis. Transcriptome sequencing results demonstrated that the transcriptional expression profile of skin tissue affected by both vitiligo and psoriasis was more similar to that of psoriasis skin tissue, further supporting the notion that psoriasis manifests on the foundation of pre‐existing vitiligo in comorbid cases. Previous immunohistochemical examinations by Ono et al. on skin tissue from a patient with psoriasis and vitiligo comorbidity revealed elevated levels of CD4, CD8, Foxp3, and IL‐17A in the comorbid skin tissue.^13^ The single‐cell transcriptional sequencing results of VP skin tissue in this study demonstrated active levels of various immune cells, suggesting that both vitiligo and psoriasis are autoimmune inflammatory diseases, and the association between them may be mediated by immune cells. Currently, it is widely believed that despite the differences in immune system stimulation between psoriasis and vitiligo, their immune pathogenesis is highly similar.^14^ Common pathways of pathogenesis include Th1/Th17 immune response, defective regulatory cells, activated LCs (Langerhans cells), CD8+ T‐cells, and tissue‐resident memory T‐cells (TRMs).^15^

The results of the Mendelian randomization study conducted in this research contradict previous observational studies and clinical observations, as it did not find evidence supporting a bidirectional causal relationship between psoriasis and vitiligo. Given the rigor of Mendelian randomization studies, it is speculated that this unexpected result may be attributed to differences in the populations studied. A multicenter study conducted in China revealed a common genetic locus in the major histocompatibility complex (MHC) region shared by psoriasis and vitiligo.^16^ However, based on a review of clinical reports worldwide, the coexistence of these two disorders appears to be coincidental.^17^ Therefore, until the differences among various populations are thoroughly investigated, it is necessary to acknowledge that, at least in the European population analyzed in this study, there is no bidirectional causality between psoriasis and vitiligo at the genetic level. Further research involving diverse populations is required to gain a comprehensive understanding of the relationship between these two conditions.

### 4.1 Strengths and limitations

This study provides valuable insights by conducting self‐control transcriptome sequencing and single‐cell transcriptome sequencing of psoriasis, vitiligo, and their comorbidities. It contributes to the existing research on these two diseases. However, it is important to acknowledge the limitations of this study. The inclusion of only one patient limits the generalizability of the results, and individual differences may introduce large errors. Mendelian randomization is a rigorous research method that aims to minimize confounding factors and errors. Although it provides valuable information, the interpretation of its results still lacks a comprehensive explanation. Mendelian randomization is only a research method based on genetic level, and the population included in this study is only a single European population, so we need to be cautious about whether the conclusions are reliable and can be extended to other populations. To further understand the relationship between psoriasis and vitiligo, researchers should consider expanding the sample size or employing new methods to conduct more systematic and scientific investigations. This would help in obtaining a more comprehensive understanding of the complex relationship between these two conditions.

## 5 CONCLUSIONS

In conclusion, the results of this study suggest that the association between vitiligo and psoriasis as an autoimmune disease is strongly immunologically related, but neither Mendelian randomization study supports causality between the two diseases in both directions. Our findings highlight the importance of considering the association between these two conditions as correlational rather than causal. These findings have implications for clinicians in terms of understanding and diagnosing psoriasis and vitiligo. However, further research is needed to fully unravel the complex relationship between psoriasis and vitiligo.

## CONFLICT OF INTEREST STATEMENT

Authors declare that there are no financial or personal relationships that could bias the work set out in the manuscript.

## ETHICS STATEMENT

This study was approved by the Ethics Committee of the Affiliated Hospital of Tianjin Academy of Traditional Chinese Medicine (approval number: LLYK2020‐21). The patients in this article have given written informed consent to the publication of their case details, data, photographs, and images of various examinations.

## COMPLIANCE STATEMENT

All experiments in this study complied with the Declaration of Helsinki and were conducted in accordance with relevant guidelines and regulations.

## Supporting information

Supporting Information

Supporting Information

Supporting Information

Supporting Information

Supporting Information

Supporting Information

## Data Availability

All data can be found in the manuscript and supplementary material.
