# Supplementary figures and images for: The relationship between psoriasis and vitiligo: From a comprehensive study
Source: Skin Res Technol. 2024 Jul 19;30(7):e13868. doi: 10.1111/srt.13868 (PMC11259540; doi:10.1111/srt.13868)

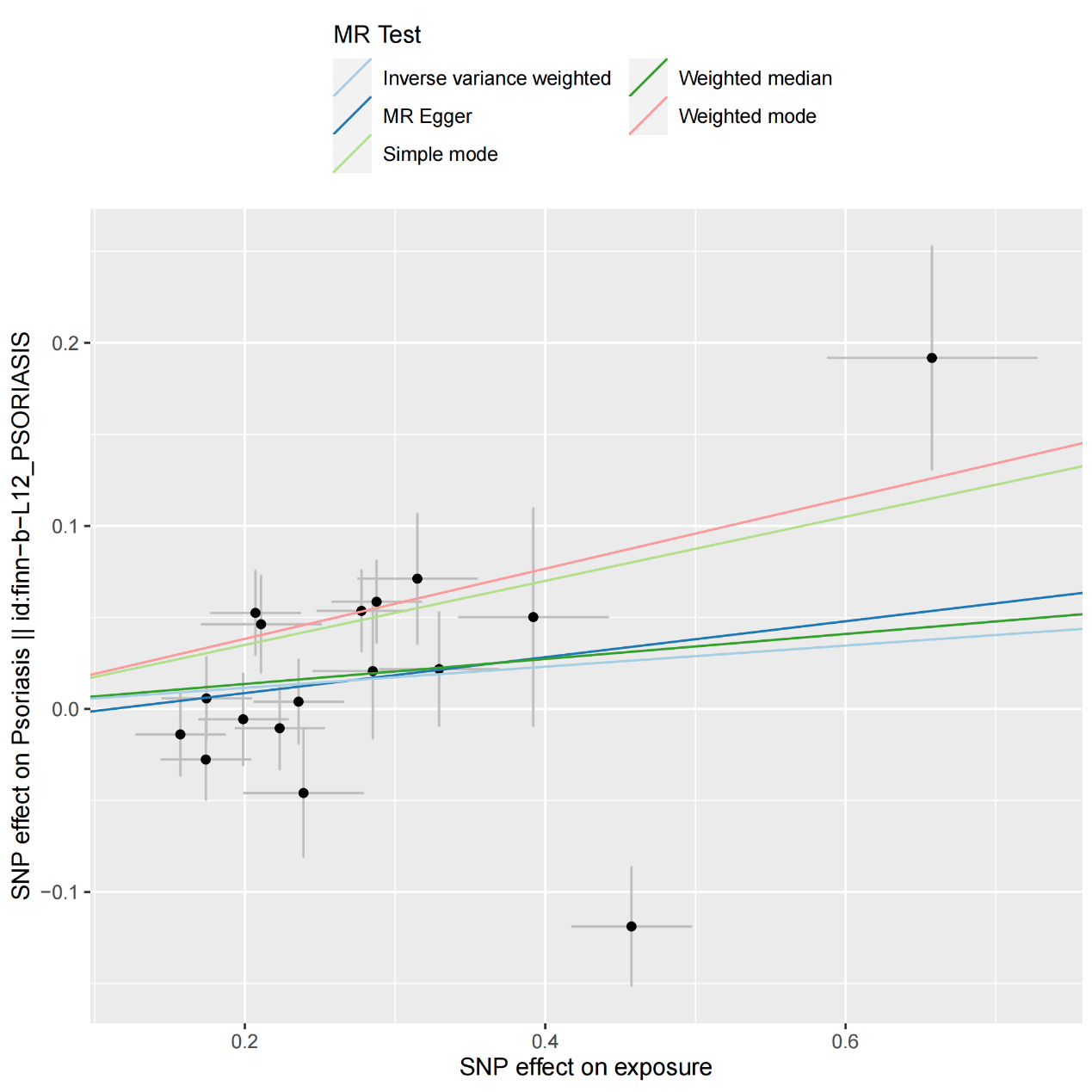


**Supplementary Figure s1 Scatter plot**

Supplement: Supplementary file 1 — Supporting Information [file SRT-30-e13868-s004.docx]

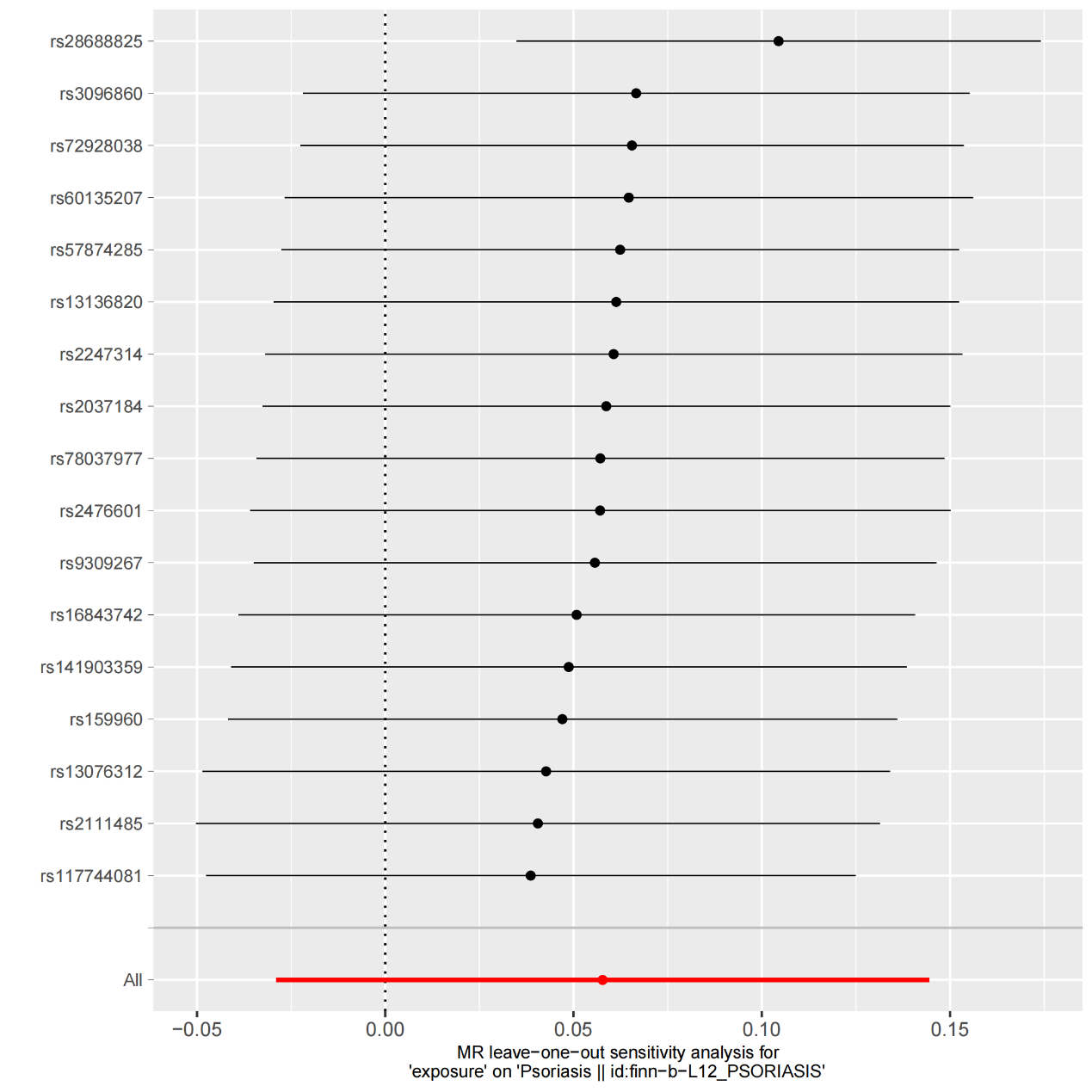


**Supplementary Figure s2 Leave-one-out plot**

Supplement: Supplementary file 2 — Supporting Information [file SRT-30-e13868-s005.docx]
