# Supplementary material for: The relationship between psoriasis and vitiligo: From a comprehensive study
Source: Skin Res Technol. 2024 Jul 19;30(7):e13868. doi: 10.1111/srt.13868 (PMC11259540; doi:10.1111/srt.13868)
Supplement: Supplementary file 3 — Supporting Information [file SRT-30-e13868-s006.docx]

[1] Wang HR, Li JG, Yang GQ, et al. Two cases of coexistence of psoriasis vulgaris and vitiligo. Chin J Clin Dermatol. 2002;31(8):488. Chinese

[2] Guo H, Zeng ZX. A case of progressive hemifacial atrophy combined with psoriasis, vitiligo, and ocular involvement. J Taishan Med Coll. 1988;1:93-94. Chiness

[3] Zhu ZY. A case report of pemphigus foliaceus associated with vitiligo and psoriasis. Shanghai Med J. 1990;6:354. Chinese

[4] Liu XM, Xie ZZ. A case of psoriasis vulgaris with vitiligo. Heilongjiang Med J. 1993;12:49. Chinese

[5] Chen L, Zheng Y. Is there a causal relationship between vitiligo and psoriasis? Chin J Dermatol Venereol. 1995;3:74. Chinese

[6] Yang YP, Liu CL, Xue K. Clinical analysis of 10 cases of psoriasis complicated by vitiligo. Mod Diagn Treat. 2013;24(13):2. Chinese

[7] Cui BN, Li L. A case of palmoplantar psoriasis with vitiligo. Chin J Dermatol Venereol. 2008;22(6):1. Chinese

[8] Liu CF, Wang LM, Liu ZZ, et al. A case of psoriasis vulgaris and thromboangiitis obliterans combined with pustular psoriasis. Chin J Dermatol. 2007;3:185. Chinese

[9] Song XY, Bu YY, Zhao Y. Three cases of psoriasis combined with vitiligo. Chin J Clin Dermatol. 2003;32(6):351. Chinese

[10] Zhu JW, Zhang XH. A report of vitiligo secondary to psoriasis. Inner Mongol J Tradit Chin Med. 2007;1:38. Chinese

[11] Tian W, Ren J, Zhang L, et al. A case of psoriasis vulgaris with vitiligo. Chin J Leprosy Skin Dis. 2005;6:487-488. Chinese

[12] Shi LL, Yang J, Qi JM. A case of psoriasis vulgaris with vitiligo. Chin J Leprosy Skin Dis. 2011;27(03):214-215. Chinese

[13] Liu S. A report of mother and daughter with coexisting vitiligo and psoriasis. Chin J Dermatol Venereol. 2002;16(2):141. Chinese

[14] Yang L, Shi SJ. A case of vitiligo associated with psoriasis vulgaris. Chin J Dermatol Venereol. 2001;15(2):134-135. Chinese

[15] Shu GY. A case of coexisting psoriasis, vitiligo, and scleroderma. Chin J Dermatol Venereol. 1999;2:46. Chinese

[16] Lang Y, Zhang R, Lang FM. A case of psoriasis vulgaris complicated by vitiligo. Chin J Dermatol Venereol. 2005;3:188. Chinese

[17] Zhang CQ, Han YP. A case of coexisting vitiligo, alopecia areata, and psoriasis vulgaris. Chin J Clin Dermatol. 1998;1:18. Chinese

[18] Wang XS, Yu CY, Zhang YG. Interferon-induced psoriasis and vitiligo: a case report. Chin J Dermatol Venereol. 2013;27(3):321. Chinese

[19] Wan P, Yang ZH, Dong TX. A case of vitiligo associated with psoriasis vulgaris. Chin J Clin Dermatol. 2002;31(5):283. Chinese

[20] Qiao SH, Geng WJ, Su JX. A report of psoriasis secondary to vitiligo. Chin J Clin Dermatol. 1996;3:29. Chinese

[21] Zheng Y, Zhang JL, Wang XY. A case of vitiligo combined with psoriasis vulgaris in the same location. Chin J Integr Tradit West Med Dermatol Section. 2019;18(1):70. Chinese

[22] An GM. A case of psoriasis vulgaris associated with vitiligo. Chin J Dermatol Venereol. 1994;3:67. Chinese

[23] Zhou PY, Wu RQ, Zhu HQ, et al. Clinical analysis of 14 cases of vitiligo complicated by psoriasis vulgaris. Chin J Clin Dermatol. 2003;32(2):78. Chinese

[24] Shang YM, Jiang F, Li JX. Treatment of psoriasis with NB-UVB irradiation combined with vitiligo and pustular psoriasis: a case report. Chin J Leprosy Skin Dis. 2006;22(12):1031. Chinese

[25] Huo YL, Nuer GL. A case of vitiligo combined with psoriasis and alopecia areata. Chin J Leprosy Skin Dis. 2010;26(6):456-457. Chinese

[26] Wang JS, Yan TK, Fang J, et al. A case of vitiligo combined with psoriasis and alopecia areata. Chin J Clin Dermatol. 2018;40(5):752-753. Chinese

[27] Lin M, Lu YH, Zhou YH, et al. Multiple autoimmune syndrome: a case of childhood alopecia areata, vitiligo, and psoriasis combined with systemic lupus erythematosus and autoimmune hepatitis.J Chin Dermatol Venereol (Electronic Edition). 2019(12):929-931. Chinese

[28] Hou J, Zhou P. A case of vitiligo associated with psoriasis and pemphigus foliaceus.Chin J Pract Med (Electronic Edition). 2001;17(6):495. Chinese

[29] Wang YP, Yang RH, Jia LZ, et al.A case of pemphigus foliaceus complicated by vitiligo and psoriasis.Chin J Leprosy Skin Dis.2010;26(12):871. Chinese

[30] Song Y, Zhang D, Xin X, et al.A case of pemphigus foliaceus combined with vitiligo and psoriasis.Chin J Clin Physician (Electronic Edition).2013;7(16):7652-7653. Chinese

[31] Yi SQ, Wang SJ, Dong L. A case of vitiligo complicated with psoriasis and allergic purpura [J]. Chinese Journal of Dermatology and Venereology, 1996, 3: 187. Chinese

[32] Ma X, Zhang ZY. A case of malignant melanoma with psoriasis and vitiligo [J]. Journal of Baotou Medical College, 1997, 3: 45-46. Chinese

[33] Liu CF, Liu HX. A case of psoriasis complicated with vitiligo and onychomycosis [J]. Chinese Journal of Leprosy and Skin Diseases, 2006, 22(9): 770. Chinese

[34] Ma ZR, He CF, Du H, et al. A case of vitiligo complicated with nevus and psoriasis [J]. Journal of Northwest National Defense Medicine, 2008, 29(3): 172. Chinese

[35] Cao Y, Wang L, Wang GP, et al. A case of multiple autoimmune syndrome - pemphigus vulgaris, vitiligo, and psoriasis complicated with Mycobacterium tuberculosis infection [J]. Chinese Journal of Dermatology, 2016, 49(4): 287-288. Chinese

[36] Hai MQ, Chao L. A case of vitiligo complicated with psoriasis and polymorphic light eruption [J]. Chinese Journal of Ethnic and Folk Medicine, 2012, 21(12): 109. Chinese

[37] Yang M, Chang JM. Two cases of plaque psoriasis complicated with vitiligo [J]. Chinese Journal of Leprosy and Skin Diseases, 2008, 24(2): 155-156. Chinese

[38] Zhao P, Wang DM. A case of psoriasis complicated with vitiligo [J]. Chinese Journal of Leprosy and Skin Diseases, 2012, 28(2): 113. Chinese

[39] Shen C. A case of vitiligo accompanied by psoriasis [J]. Road to Health, 2017, 16(12): 245. Chinese

[40] Chen Y, Guo ZP, Li W. A case of vitiligo complicated with psoriasis [J]. Journal of Clinical Dermatology, 2003, 31(3): 161. Chinese

[41] Li ZH, Tang L, Mao SH. A case of vitiligo associated with plaque psoriasis [J]. Chinese Journal of Integrated Traditional and Western Medicine for Skin Diseases, 2004, 3(2): 119. Chinese

[42] Liu AP, Yan XY. A case of psoriasis complicated with vitiligo [J]. Chinese Community Physicians (Comprehensive Edition), 2007, 1: 55. Chinese

[43] Yu ML, Shi ZX, Zhang JR, et al. Report of two cases of plaque psoriasis combined with vitiligo [J]. Dermatology and Venereology (Skin Diseases and Sexually Transmitted Diseases), 2000, 1: 50. Chinese

[44] Chen XF, Song DY. A case of vitiligo complicated with psoriasis [J]. Journal of Clinical Dermatology, 2002, 5: 311. Chinese

[45] Sun HW, Yang GL, Du H, et al. A case of concurrence of psoriasis with the mother and vitiligo with the father [J]. Chinese Journal of Dermatology and Venereology, 2007, 21(10): 639-640. Chinese
