# Supplementary material for: The relationship between psoriasis and vitiligo: From a comprehensive study
Source: Skin Res Technol. 2024 Jul 19;30(7):e13868. doi: 10.1111/srt.13868 (PMC11259540; doi:10.1111/srt.13868)
Supplement: Supplementary file 4 — Supporting Information [file SRT-30-e13868-s003.docx]

| Table s1. Details of instrumental variables for vitiligo | | | | | | | | | | | | |
| --- | --- | --- | --- | --- | --- | --- | --- | --- | --- | --- | --- | --- |
| SNP | Chromosome | Position | A1 | A2 | Beta.exposure | SE.exposure | Pval.exposure | Beta.outcome | SE.outcome | Pval.outcome | EAF | F-statistics |
| rs117744081 | 7 | 29132279 | G | A | 0.657520003 | 0.07 | 1.62E-21 | 0.1918 | 0.0613 | 0.001749 | 0.03404 | 88.23113352 |
| rs13076312 | 3 | 188089254 | T | C | 0.277631737 | 0.03 | 4.63E-22 | 0.0536 | 0.0225 | 0.0169801 | 0.526 | 85.64375685 |
| rs13136820 | 4 | 40307564 | T | C | -0.198850859 | 0.03 | 1.72E-11 | 0.0056 | 0.0253 | 0.8259 | 0.7347 | 43.93518225 |
| rs141903359 | 3 | 119283543 | G | A | 0.31481074 | 0.04 | 6.89E-15 | 0.0712 | 0.0358 | 0.0467897 | 0.1087 | 61.9411262 |
| rs159960 | 1 | 8476428 | A | G | 0.207014169 | 0.03 | 8.08E-13 | 0.0525 | 0.0233 | 0.0244399 | 0.3595 | 47.61651814 |
| rs16843742 | 1 | 198672299 | C | T | -0.210721031 | 0.04 | 6.11E-09 | -0.0463 | 0.0267 | 0.083029 | 0.2289 | 27.75209565 |
| rs2037184 | 3 | 187795657 | C | T | -0.174353387 | 0.03 | 4.24E-09 | -0.0058 | 0.0229 | 0.8011 | 0.4261 | 33.77678179 |
| rs2111485 | 2 | 163110536 | G | A | 0.287682072 | 0.03 | 2.69E-22 | 0.0586 | 0.0227 | 0.00981409 | 0.5822 | 91.95663868 |
| rs2247314 | 6 | 167370230 | C | T | -0.235722334 | 0.03 | 1.30E-13 | -0.004 | 0.0233 | 0.8633 | 0.3711 | 61.73890947 |
| rs2476601 | 1 | 114377568 | A | G | 0.329303747 | 0.04 | 2.21E-14 | 0.0218 | 0.0314 | 0.4863 | 0.1487 | 67.77559868 |
| rs2687812 | 8 | 133931055 | T | A | -0.19062036 | 0.03 | 1.98E-11 | 0.0111 | 0.0228 | 0.625801 | 0.6034 | 40.37346833 |
| rs28688825 | 6 | 32587157 | G | A | 0.457424847 | 0.04 | 1.76E-38 | -0.1188 | 0.0327 | 0.000274202 | 0.1436 | 130.7734317 |
| rs3096860 | 2 | 204786162 | C | T | 0.173953307 | 0.03 | 1.02E-08 | -0.0276 | 0.0224 | 0.2192 | 0.4718 | 33.62194784 |
| rs57874285 | 2 | 112012672 | A | C | 0.157003749 | 0.03 | 4.56E-08 | -0.0139 | 0.0229 | 0.5452 | 0.3918 | 27.38908571 |
| rs60135207 | 3 | 71563777 | T | G | -0.223143551 | 0.03 | 2.44E-14 | 0.0105 | 0.0229 | 0.6454 | 0.394 | 55.32560499 |
| rs7007905 | 8 | 134209453 | G | C | -0.186329578 | 0.03 | 7.28E-09 | -0.0343 | 0.0269 | 0.2016 | 0.2257 | 38.57634634 |
| rs72928038 | 6 | 90976768 | A | G | 0.2390169 | 0.04 | 1.42E-11 | -0.0459 | 0.0352 | 0.1917 | 0.1147 | 35.70567419 |
| rs78037977 | 1 | 172715702 | G | A | 0.285178942 | 0.04 | 1.86E-13 | 0.0207 | 0.0371 | 0.5768 | 0.1028 | 50.82939318 |
| rs9309267 | 2 | 55850333 | T | C | 0.392042088 | 0.05 | 5.46E-13 | 0.0502 | 0.0597 | 0.4003 | 0.03593 | 61.47879944 |
