# Supplementary material for: The relationship between psoriasis and vitiligo: From a comprehensive study
Source: Skin Res Technol. 2024 Jul 19;30(7):e13868. doi: 10.1111/srt.13868 (PMC11259540; doi:10.1111/srt.13868)
Supplement: Supplementary file 5 — Supporting Information [file SRT-30-e13868-s002.docx]

| Table s2. Details of instrumental variables for psoriasis | | | | | | | | | | | | |
| --- | --- | --- | --- | --- | --- | --- | --- | --- | --- | --- | --- | --- |
| SNP | Chromosome | Position | A1 | A2 | Beta.exposure | SE.exposure | Pval.exposure | Beta.outcome | SE.outcome | Pval.outcome | EAF | F-statistics |
| rs12188300 | 5 | 158829527 | T | A | 0.4331 | 0.0495 | 2.24E-18 | 0 | 0.05 | 0.968 | 0.05898 | 76.55366187 |
| rs12713428 | 2 | 61118113 | C | A | 0.1694 | 0.0261 | 8.11E-11 | -0.030459207 | 0.03 | 0.426 | 0.2487 | 42.12557068 |
| rs17728338 | 5 | 150478318 | A | G | 0.3092 | 0.0439 | 1.76E-12 | -0.083381609 | 0.06 | 0.19 | 0.07331 | 49.60779573 |
| rs60600003 | 7 | 37382465 | G | T | 0.2128 | 0.0372 | 1.03E-08 | -0.030459207 | 0.05 | 0.494 | 0.1017 | 32.72332062 |
| rs674451 | 6 | 138216788 | C | T | 0.1307 | 0.0235 | 2.82E-08 | -0.010050336 | 0.03 | 0.783 | 0.343 | 30.93253056 |
| rs9481169 | 6 | 111929862 | T | G | 0.2515 | 0.0422 | 2.47E-09 | 0.076961041 | 0.05 | 0.113 | 0.07817 | 35.51821051 |
|  |  |  |  |  |  |  |  |  |  |  |  |  |
|  |  |  |  |  |  |  |  |  |  |  |  |  |
