# Supplementary material for: The relationship between psoriasis and vitiligo: From a comprehensive study
Source: Skin Res Technol. 2024 Jul 19;30(7):e13868. doi: 10.1111/srt.13868 (PMC11259540; doi:10.1111/srt.13868)
Supplement: Supplementary file 6 — Supporting Information [file SRT-30-e13868-s001.docx]

| Table s3. Heterogenity and pleiotropy analyses | | | | | |
| --- | --- | --- | --- | --- | --- |
|  | Heterogenity | | MR-Egger intercept | | |
|  | Q | Q_pval | egger_intercept | se | pval |
| Forward (vitiligo-psoriasis) | 47.728 | 0.000 | -0.011 | 0.037 | 0.770 |
| Reverse (psoriasis-vitiligo) | 5.113 | 0.276 | -0.013 | 0.079 | 0.881 |
